# Cost-effectiveness of Apixaban for Stroke Prevention in Patients with Atrial Fibrillation in Algeria

**DOI:** 10.36469/9797

**Published:** 2017-05-05

**Authors:** Yazid Aoudia, Thitima Kongnakorn, Evie Merinopoulou, Mohamed Said Bettayeb, Sid Ahmed Kherraf

**Affiliations:** 1 Department of Cardiology A1 Mustafa Hospital, Place du 1er Mai 1945, Sidi M’Hamed, 16000, Algiers, Algeria; 2 Evidera, Metro Building, 6th Floor, 1 Butterwick, London, W6 8DL UK; 3 Pfizer Algeria, Algeria Business Center Pins Maritimes 16211, Algiers, Algeria; 4 Pfizer Gulf, Pfizer building, Dubai Media City, PO Box 502749 - Dubai, United Arab Emirates

**Keywords:** algeria, aspirin, rivaroxaban, acenocoumarol, atrial fibrillation, cost-effectiveness, apixaban

## Abstract

**Background:** Atrial fibrillation (AF) is a chronic sustained heart rhythm disorder associated with an increased risk of stroke. Apixaban, a new oral anticoagulant, was approved by the European Medicines Agency for prevention of stroke in patients with AF. The efficacy of apixaban has been investigated in randomised controlled trials.

**Objectives:** The objective of this study was to estimate the economic implications of using apixaban compared to other anti-coagulations to reduce the risk of stroke in patients with AF from the perspective of the Algerian payer.

**Methods:** A previously published Markov model was adapted to the Algerian setting. The model included patients for whom vitamin K antagonist (VKA) treatment is suitable and could initiate on acenocoumarol, rivaroxaban or apixaban, and those unsuitable for VKA treatment who could initiate on aspirin or apixaban. Over a lifetime time horizon, costs were estimated in Algerian dinars (DZD) and outcomes included life-years (LYs), quality-adjusted life-years (QALYs) and incremental cost-effectiveness ratios (ICERs).

**Results:** In the VKA suitable population, apixaban was estimated to be a dominant treatment option over rivaroxaban, providing a higher number of QALYs at lower costs, while when compared with acenocoumarol, an ICER of 3 672 059 DZD per QALY gained was estimated. Amongst those unsuitable for VKA therapy, the ICER was 2 061 863 DZD per QALY gained.

**Conclusion:** Apixaban was found to be a cost-effective choice for stroke prevention in patients with AF in Algeria compared to acenocoumarol and rivaroxaban in the VKA suitable population and compared to aspirin in the VKA unsuitable population.

## BACKGROUND

Atrial fibrillation (AF) is a chronic sustained heart rhythm disorder most common among the elderly.[1] The burden of AF at age 65 years is nearly seven times that at age 45 years, and only one-quarter that at age 80 and older.[2] Patients with AF have a five-fold increased risk of stroke, and it is estimated that up to 25% of all strokes in the elderly are a consequence of AF.[1] Aging populations suffer from an increasing morbidity, mortality and economic burden of non-communicable diseases such as cardiovascular disease, AF and stroke. A rapid 11% increase in life expectancy seen since 1990 in the North Africa and Middle East (NAME) region, including Algeria, contributed to the modest increase of the prevalence of AF (2.6% [uncertainty intervals: -24.5 to 42.2] median change between 1990 and 2010) and stroke (5.6% between 2000 and 2012).[2,3] Based on the projection of the World Health Organization (WHO), stroke-related mortality in the NAME region is expected to increase by 23% between 2015 and 2030.[4] Overall, health spending per capita in the NAME region is estimated to be 956 and 932 United States dollar (USD) (PPP, 2005), respectively.[3]

According to the 2016 European Society of Cardiology (ESC) Guidelines for the management of AF, when oral anticoagulation is initiated in a patient with AF who is eligible for a non-vitamin K antagonist oral anticoagulant (NOAC) drugs (apixaban, dabigatran, edoxaban, or rivaroxaban), a NOAC is recommended in preference to a vitamin K antagonist. Antiplatelet monotherapy is not recommended for stroke prevention in patients with AF, regardless of stroke risk.[5] In a recently published international cross-sectional survey on the use of antithrombotic therapy in patients with AF, the results showed that patients with the greatest risk of stroke (i.e., $CHADS_2 \geq 2$) in the Middle East and Africa region had the highest oral anticoagulant use (66.7%).[6]

Apixaban is a direct and highly selective active site inhibitor of factor Xa that has been approved by the European Medicines Agency for stroke prevention in patients with AF. The efficacy and safety of apixaban (5 mg twice daily [b.i.d]) versus dose-adjusted warfarin and aspirin has been studied in two large randomised, multicentre, double-blind, Phase III trials (ARISTOTLE[7] and AVERROES,[8] respectively) in patients with non-valvular atrial fibrillation (NVAF) and one or more additional risk factors (i.e., prior stroke, age, symptomatic heart failure, hypertension and/or diabetes mellitus). Apixaban was found to significantly reduce the risk of stroke and systemic embolism, major bleeding and all-cause mortality compared to warfarin. In patients who were unsuitable for vitamin K antagonist therapy, apixaban significantly reduced the risk of stroke and systemic embolism without significantly increasing the risk of major bleeding compared with aspirin.

An assessment of the cost-effectiveness of available therapies for stroke prevention in patients with AF has not been previously performed in Algeria. To inform decision making around optimal treatment, it is important to consider the costs and benefits for alternative therapies. To this end, the objective of this study was to estimate the economic implications of using apixaban compared to other anti-coagulations to reduce the risk of stroke in patients with AF from the perspective of the Algerian payer.

## METHODS

### Model Design

A previously developed and validated Markov model9,10 was used to evaluate the long-term clinical and economic outcomes in Algerian patients with AF receiving anticoagulant treatment over a lifetime. This evaluation was conducted from the perspective of the Algerian payer. The model included two cohorts of patients – those suitable for VKA treatment and those unsuitable for VKA treatment. VKA-suitable patients could initiate on either of the following treatments: dose-adjusted acenocoumarol, rivaroxaban 20 mg once daily or apixaban 5 mg b.i.d. Patients who were VKA unsuitable could initiate on aspirin or apixaban. Patients transitioned through health states including: NVAF, ischaemic stroke and haemorrhagic strokes (mild, moderate, severe and fatal), intracranial haemorrhage (ICH) other than haemorrhagic strokes (referred to as other ICH), systemic embolism, myocardial infarction, other major bleeds (OMB; non-ICH major bleeds), clinically relevant non-major (CRNM) bleeds and NVAF with subsequent aspirin treatment or death (Figure 1).

### Model Inputs

Patient population characteristics including age, gender and $CHADS_2$ distribution, as well as clinical event rates, were taken from the ARISTOTLE and AVERROES trials.[7,8] Event rates for acenocoumarol were assumed the same as warfarin, obtained from the ARISTOTLE trial. For rivaroxaban, clinical event rates were taken from indirect treatment comparison in the absence of head-to-head clinical trial data.[9,10] Tables 1 and 2 show a summary of inputs included in the model.

**Table 1.** Population Demographic and Clinical Characteristics

| | VKA Unsuitable | VKA Suitable | Source |
|---|---|---|---|
| Starting age (mean, years) | 70 | 70 | 7,8 |
| Gender (% male) | 58.5 | 64.7 | 7,8 |
| $CHADS_2$ distribution (%) | | | 7,8 |
| 0–1 | 38.2 | 34 | |
| 2 | 35.2 | 35.8 | |
| ≥ 3 | 26.6 | 30.2 | |
| Average $CHADS_2$ score | 2.0 | 2.1 | 7,8 |

**Table 2.** Clinical Event Rates by Treatment

| | VKA Unsuitable | | | VKA Suitable | | | |
|---|---|---|---|---|---|---|---|
| | Apixaban | Aspirin | Source | Apixaban | Acenocoumarol* | Rivaroxaban | Source |
| Event | Rate of events per 100 patient years | | | | | HR vs. apixaban (95% CI) | |
| Ischaemic stroke | 1.37 | 3.10 | 9 | 0.98 | 1.08 | 0.98 (0.72, 1.33) | 9,10 |
| Intracranial haemorrhage | 0.34 | 0.35 | 9 | 0.33 | 0.80 | 1.73 (1.08, 2.77) | 9,10 |
| Other major bleed | 1.07 | 0.57 | 9 | 1.79 | 2.27 | 1.46 (1.15, 1.79) | 9,10 |
| Clinically relevant non-major bleed | 3.11 | 2.37 | 9 | 2.08 | 2.99 | 1.49 (1.26, 1.76) | 9,10 |
| Myocardial infarction | 0.76 | 0.89 | 9 | 0.53 | 0.61 | 0.94 (0.64, 1.38) | 9,10 |
| Systemic embolism | 0.06 | 0.41 | 9 | 0.09 | 0.10 | 1.00 | 9,10 |
| Other cardiovascular hospitalisation | 10.46 | 12.09 | 9 | 10.46 | 10.46 | 1.00 | 9,10 |
| Other treatment discontinuation | 17.31 | 19.01 | 9 | 13.18 | 14.41 | 1.18 (1.08, 1.29) | 9,10 |
| Other death | 2.97 | 3.59 | 9 | 3.08 | 3.34 | 1.00 | 9,10 |

CI: confidence interval; HR: hazard ratio; VKA: vitamin K antagonist
* Clinical event rates in this analysis are assumed to be the same as warfarin from ARISTOTLE trial.

**Figure 1.** Schematic Representation of the Markov Model

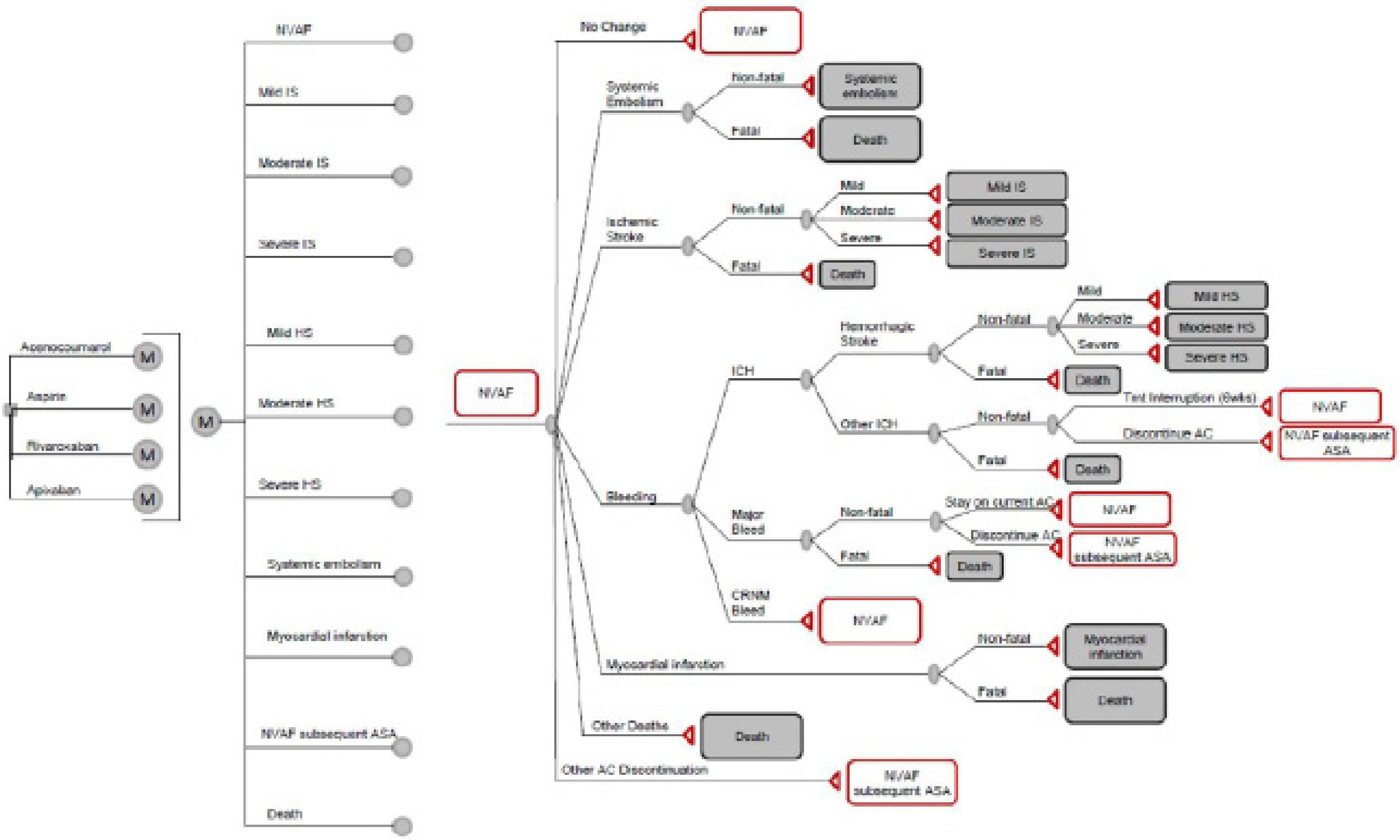

Abbreviations: AC, anti-coagulant; ASA, aspirin; CRNM, clinically relevant non-major; HS, haemorrhagic stroke; ICH, intracranial haemorrhage; IS, ischaemic stroke; MI, myocardial infarction; NVAF, non valvular atrial fibrillation; SE, systemic embolism; Tmt

Background mortality estimates, resource use and cost inputs were adapted to the Algerian setting. Background mortality was derived from age- and gender-specific life tables of the Algerian population taken from WHO.[11] Direct costs in Algerian dinars (DZD), at 2015 prices, specific to the Algerian market were applied to the model (Table 3). The cost estimates of clinical events were obtained from a local Algerian private clinic and the university hospital CHU Mustapha. Health and cost outcomes were discounted at an annual rate of 3.5% as in prior published studies in the United Kingdom (UK).[9,10]

**Table 3.** Model Cost Inputs Adapted to the Algerian Market

| **Anticoagulation Costs (per tablet)** | | | **Source** |
|---|---|---|---|
| Apixaban | 193.51 DZD | | Local market |
| Aspirin (first line) | 5.88 DZD | | Local market |
| Acenocoumarol | 5.50 DZD | | Local market |
| Rivaroxaban | 455.00 DZD | | Local market |
| **Health state** | **Acute care (per episode)*** | **Long-term cost (per month)** | **Source** |
| Ischaemic stroke | | | |
| Mild | 143 000 DZD | 18 000 DZD | Local private clinic |
| Moderate | 293 000 DZD | 32 000 DZD | Local private clinic |
| Severe | 543 000 DZD | 48 000 DZD | Local private clinic |
| Fatal | 743 000 DZD | | Local private clinic |
| Haemorrhagic stroke | | | |
| Mild | 323 000 DZD | 18 000 DZD | Local private clinic |
| Moderate | 573 000 DZD | 32 000 DZD | Local private clinic |
| Severe | 810 000 DZD | 48 000 DZD | Local private clinic |
| Fatal | 935 000 DZD | | Local private clinic |
| Myocardial infarction | 42 000 DZD | 4833 DZD | CHU Mustapha |
| Systemic embolism | 39 000 DZD | 10 000 DZD | Local private clinic |
| **Bleeding & other CV hospitalisation** | **Acute care (per episode)** | | **Source** |
| Other ICH (excl. haemorrhagic stroke) | 350 000 DZD | | CHU Mustapha |
| GI bleeds (per episode) | 76 500 DZD | | CHU Mustapha |
| Non ICH and non-GI-related major bleeds (per episode) | 60 000 DZD | | CHU Mustapha |
| CRNM bleeds (per episode) | 25 000 DZD | | CHU Mustapha |
| Other CV hospitalisation (per episode) | 200 000 DZD | | CHU Mustapha |
| **Management Costs** | | | **Source** |
| Dyspepsia (annually) | 8300 DZD | | Local GI specialist |
| Renal monitoring (per test) | 1200 DZD | | Local laboratory |
| Monitoring visit (applicable to acenocoumarol only) (per visit) | 200 DZD | | Local laboratory |
| Routine care (per visit) | 1500 DZD | | Local expert |

CRNM: clinically relevant non-major; CV: cardiovascular; GI: gastrointestinal; ICH: intracranial haemorrhage; CHU Mustapha: centre hospitalier universitaire- university hospital Mustapha

*Acute period for strokes and HS (mild, moderate and severe) were to be 2 weeks.

Patients were assigned utilities according to their health state (where a utility of 1 denotes full health and 0 denotes death) as presented in Table 4. In the absence of Algerian-specific utility estimates, the model utilised estimates from a UK-based EuroQol-5 dimensions (EQ-5D) catalogue as those used in earlier models.[9,10,12] Utility decrements associated with the use of treatments were applied, with the highest disutility applied to acenocoumarol due to monitoring and drug interactions based on prior warfarin studies.[13]

**Table 4.** Model Inputs on Utilities

| Health State | Utility (standard error) | Source |
|---|---|---|
| Non-valvular atrial fibrillation | 0.7270 (0.0095) | 12 |
| Stroke or haemorrhagic stroke | | |
| Mild | 0.6151 (0.0299) | 12 |
| Moderate | 0.5646 (0.0299) | 12 |
| Severe | 0.5142 (0.0299) | 12 |
| Myocardial infarction | | |
| Females | 0.6151 (0.0299) | 12 |
| Males | 0.5646 (0.0299) | 12 |
| Systemic embolism | 0.6265 (0.0299) | 12 |
| **Transient health states/anticoagulation use** | **Utility decrement (standard error/95% CI)** | |
| Other intracranial hemorrhage | 0.1511 (0.0401) | 12 |
| Other major bleeds | 0.1511 (0.0401) | 12 |
| Clinically relevant non-major bleed | 0.0582 (0.0173) | 12 |
| Other cardiovascular hospitalisation | 0.1276 (0.0259) | 12 |
| Apixaban or aspirin | 0.0020 (0.00–0.04) | 13 |
| Acenocoumarol* | 0.0120 (0.00–0.08) | 13 |

CI: onfidence interval
* Utility decrements in this analysis are assumed to be the same as warfarin.

### Analyses

Apixaban was compared with acenocoumarol and rivaroxaban among the VKA-suitable population. Among the VKA-unsuitable population, apixaban was compared with aspirin. For a cohort of 1,000 patients, the model estimated the total clinical benefit in terms of number of clinical events, estimated life-years (LYs), quality-adjusted life-years (QALYs), as well as costs, over a lifetime time horizon for each treatment. The relative clinical benefit of apixaban compared to other treatments was assessed using incremental cost-effectiveness ratios (ICERs), which denoted the cost per QALY gained per average patient for the adoption of apixaban over the comparator treatments.

In addition to the analyses using the primary inputs – known as base-case – univariate sensitivity analyses were conducted to explore the impact of various input parameters on the ICERs including: varying discount rates, event risks for each treatment, the HRs for acenocoumarol, rivaroxaban, and aspirin versus apixaban, as well cost and utility inputs. These input parameters were varied by their 95% confidence intervals where available. Scenario analyses were also conducted to examine the impact of key assumptions in the model. The evaluated scenarios included: 1) variation in the $CHADS_2$ distribution, 2) variation in the quality of international normalised ratio (INR) control (i.e., the centre time in therapeutic range [cTTR] distribution), 3) variations in population characteristics and INR control estimated for the Algerian population, 4) assumptions on second-line treatment (i.e., no treatment), and 5) assumptions on treatment discontinuation (i.e., 0 risk of discontinuation, or same as apixaban).

Furthermore, probabilistic sensitivity analyses were conducted by running 2000 iterations of a cohort of 1000 patients, with the model parameters drawn randomly from probability distributions in each iteration. The results of the probabilistic sensitivity analyses were presented as a scatterplot of the incremental QALYs versus incremental costs for apixaban versus comparators in both the VKA-suitable and VKA-unsuitable populations. A cost-effectiveness acceptability curve (CEAC) was generated representing the proportion of the iterations for which each treatment was considered the most cost-effective alternative at a given threshold of willingness to pay for a QALY gained. Given no established thresholds exist in Algeria, the analysis assessed the cost-effectiveness of apixaban versus each of the comparators using threshold values commonly used in the United States ($50 000/QALY equal to DZD 5 430 721, based on exchange rates in 2015 where $1=DZD 108.6), and the UK (£30 000/QALY equal to DZD 4 779 190, based on exchange rates in 2015 where £1=DZD 159.3).

## RESULTS

### Base Case

Among a cohort of 1000 patients with AF who were VKA-unsuitable, patients treated with apixaban were predicted to experience 58 fewer strokes than patients treated with aspirin. The number of bleeds increased in patients treated with apixaban, with 32 additional major bleeds (haemorrhagic strokes, OMB and ICHs) and 56 additional CRNM bleeds compared to patients treated with aspirin (Table 5). The reduction in strokes associated with apixaban treatment translated into 0.205 additional discounted QALYs and 0.237 additional discounted LYs.

In the cohort of VKA-suitable patients with AF, those treated with apixaban were predicted to experience 43 fewer major bleeds (haemorrhagic strokes, OMB and ICHs) than patients treated with acenocoumarol or rivaroxaban. CRNM bleeds were also reduced by 41 and 52 events compared to acenocoumarol and rivaroxaban, respectively (Table 5). The reduction in clinical events among patients treated with apixaban was associated with 0.14 and 0.05 additional discounted LYs, and an additional 0.14 and 0.04 discounted QALYs compared to acenocoumarol and rivaroxaban, respectively.

Treatment costs are presented by event costs, anticoagulant treatment and management costs, routine care and monitoring in Table 5. In the VKA-unsuitable population, the model predicted an average lifetime discounted total cost per patient of 920 612 DZD for patients treated with apixaban, and a lifetime discounted cost of 496 716 DZD for patients treated with aspirin. Among the VKA-suitable population, the average lifetime discounted total cost per patient was 978 530 DZD for patients treated with apixaban; lifetime discounted costs for patients treated with acenocoumarol and rivaroxaban were 441 714 DZD and 1 031 387 DZD respectively. The ICER of apixaban versus aspirin in the VKA-unsuitable population was 2 061 863 DZD ($20 866, £12 943; based on exchange rates in 2015, where $1=DZD 108.6 and £1=DZD 159.3) per QALY gained. Treatment with apixaban in the VKA-suitable population dominated rivaroxaban by having higher incremental QALYs at lower costs. Compared to acenocoumarol, the resulting ICER was 3 672 059 DZD ($37 054, £23 051; based on exchange rates in 2015, where $1=DZD 108.6 and £1=DZD 159.3) per QALY gained.

**Table 5.** Base-case results over lifetime: clinical events predicted among 1 000 patients, mean LYs, QALYs and costs per patient

| | VKA Unsuitable | | VKA Suitable | | |
|---|---|---|---|---|---|
| **Variable** | **Apixaban** | **Aspirin** | **Apixaban** | **Acenocoumarol** | **Rivaroxaban** |
| **No. of events (per cohort of 1000)** | | | | | |
| Strokes* | 206 | 264 | 183 | 187 | 190 |
| Haemorrhagic strokes* | 16 | 15 | 23 | 37 | 26 |
| Intracranial haemorrhage | 13 | 12 | 10 | 22 | 19 |
| Systemic embolism | 18 | 29 | 18 | 19 | 18 |
| Other major bleed | 87 | 58 | 143 | 161 | 173 |
| Clinically relevant non-major bleed | 266 | 210 | 225 | 266 | 277 |
| Myocardial infarction | 75 | 75 | 65 | 68 | 67 |
| Cardiovascular hospitalisation | 935 | 925 | 947 | 928 | 946 |
| Other treatment discontinuation | 660 | 640 | 588 | 593 | 625 |
| **Death** | | | | | |
| Event related | 307 | 369 | 281 | 305 | 296 |
| Other | 693 | 631 | 718 | 695 | 704 |
| **Health outcomes (per patient)** | | | | | |
| LYs (discounted) | 7.146 | 6.909 | 7.273 | 7.137 | 7.219 |
| QALYs (discounted) | 5.099 | 4.894 | 5.199 | 5.053 | 5.157 |
| **Costs (DZD discounted per patient)** | | | | | |
| Anticoagulant and management | 517 857 | 22 058 | 591 275 | 20 289 | 630 429 |
| Monitoring | 764 | 1868 | 668 | 12 191 | 747 |
| Routine care | 38 570 | 37 297 | 39 251 | 38 508 | 38 954 |
| Clinical events | 363 421 | 435 493 | 347 335 | 370 726 | 361 257 |
| **Total** | 920 612 | 496 716 | 978 530 | 441 714 | 1 031 387 |
| **Incremental cost-effectiveness ratio (apixaban versus comparator) DZD per QALY gained** | | 2 061 863 | | 3 672 059 | Dominant |

LY: life-year; QALY: quality-adjusted life-year
*Denotes first and recurrent stroke events.

## Univariate Sensitivity Analyses

Results from univariate sensitivity analyses are presented as tornado diagrams (Figures 2a-2b). The ICERs of apixaban compared to aspirin in VKA unsuitable population varied from DZD 1 235 054 ($11 373, £7753; based on exchange rates in 2015, where $1=DZD 108.6 and £1=DZD 159.3) to DZD 3 557 026 ($32 753, £22 329; based on exchange rates in 2015, where $1=DZD 108.6 and £1=DZD 159.3) per QALY, with the most influential parameters being ischemic stroke risk for aspirin and apixaban and the cost of apixaban (Figure 2a). While for the comparison of apixaban to acenocoumarol in VKA suitable population, the ICERs varied from DZD 1 328 832 ($12 236, £8342; based on exchange rates in 2015, where $1=DZD 108.6 and £1=DZD 159.3) to DZD 5 581 883 ($51 399, £35 040; based on exchange rates in 2015, where $1=DZD 108.6 and £1=DZD 159.3) per QALY. The most influential parameters included the assumption around utility decrement associated with use of acenocoumarol, and the risk of death for apixaban and acenocoumarol during the trial period (Figure 2b).

In comparison with rivaroxaban, apixaban was dominant in all scenarios, except for the assumption on the cost of apixaban and cost of rivaroxaban with upper ICER estimates ranging from 1 502 931 ($13 839, £9435) DZD to 1 684 230 DZD ($15 509, £10 573) per QALY.

**Figure 2a.** Tornado Chart for Apixaban versus Aspirin in the VKA-unsuitable Population (ICER)*

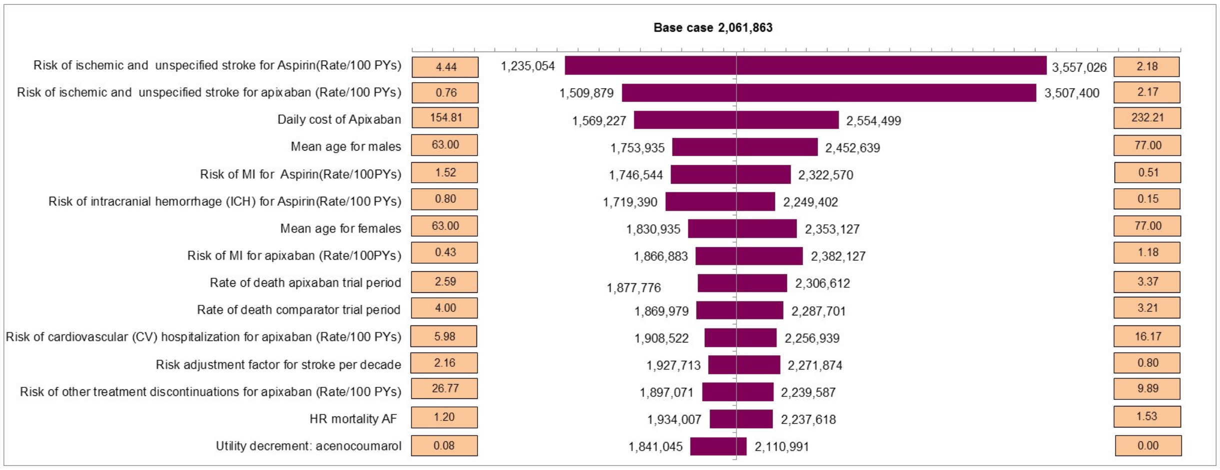

**Figure 2b.** Tornado Chart for Apixaban versus Acenocoumarol in the VKA-suitable Population (ICER)*

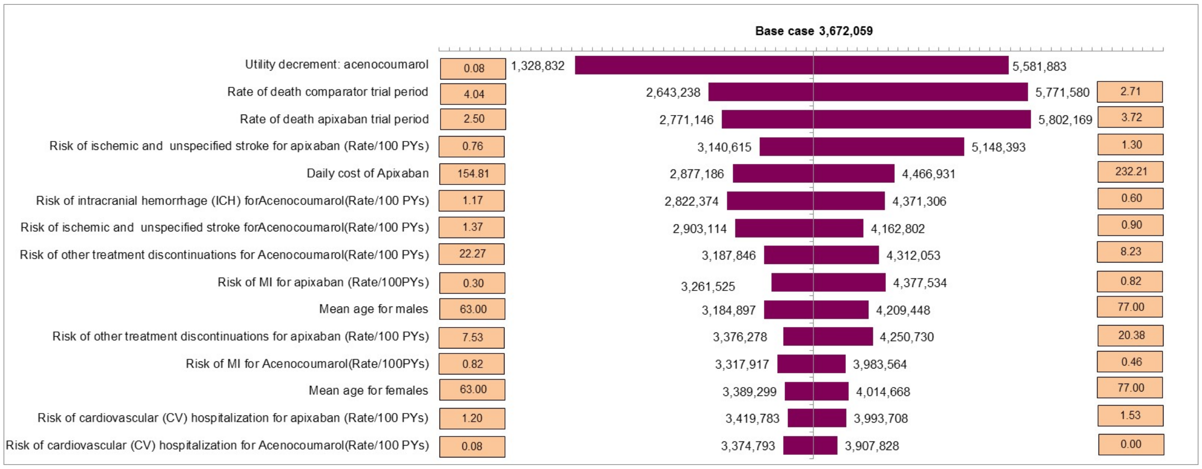

*Top 15 parameters that the ICERs are most sensitive to (DZD/QALY).
ICER: incremental cost-effectiveness ratio; VKA: vitamin K antagonist

### Scenario Analyses

The results from the scenario analyses are detailed in Table 6. These showed that the ICER for the comparison of apixaban to aspirin in the VKA unsuitable population was mostly sensitive in low-risk patients (i.e., those with $CHADS_2$ score less than 1) with the least favourable ICER (97.53% increase from base case) whereas in the VKA suitable population, results were mostly sensitive to the quality of INR control cTTR distribution, with the most favourable ICER (33.02% decrease from base case) in patients with poorly control INR (i.e., cTTR < 52.38%). In the VKA-suitable population, in all scenarios tested, apixaban dominated rivaroxaban, providing higher number of QALYs at lower costs.

**Table 6.** Scenario Analyses

| Scenario | Values | VKA Unsuitable: ICER vs Aspirin (% deviation from base case) | VKA Suitable: ICER vs Acenocoumarol (% deviation from base case) |
|---|---|---|---|
| Base | | 2 061 863 | 3 672 059 |
| $CHADS_2$ distribution | $CHADS_2$ = 0-1: 100% | 4 072 745 (97.53) | 4 145 089 (12.88) |
| | $CHADS_2$ = 2: 100% | 1 986 506 (-3.65) | 4 080 614 (11.13) |
| | $CHADS_2$ ≥ 3: 100% | 1 226 578 (-40.51) | 3 097 757 (-15.64) |
| Quality of INR control cTTR distribution (ranges) | cTTR < 52.38%: 100% | | 2 459 483 (-33.02) |
| | 52.38% ≤ cTTR < 66.02%: 100% | | 5 153 239 (40.34) |
| | 66.02% ≤ cTTR < 76.51%: 100% | | 4 764 296 (29.74) |
| | cTTR ≥ 76.51%: 100% | | 3 510 949 (-4.39) |
| Algerian cTTR | cTTR < 52.38%: 61%; 52.38% ≤ cTTR < 66.02%: 3%; 66.02% ≤ cTTR < 76.51%: 6%; cTTR ≥ 76.51%:31% | | 2 845 123 (-22.52) |
| Age, gender and $CHADS_2$ distribution | Algerian distribution: Gender: 64.7% male; Mean age: 70 years (both males/females) $CHADS_2$ = 0-1: 36.7%; $CHADS_2$ = 2: 35.0%; $CHADS_2$ ≥ 3: 28.3% | 3 893 680 (88.57) | 3 754 142 (2.24) |
| Second-line treatment | No treatment | 2 117 727 (2.71) | 3 405 412 (-7.26) |
| Treatment discontinuation | Set risk of discontinuation to 0 | 2 252 549 (9.25) | 3 859 797 (5.11) |
| | Same as apixaban | 2 051 604 (-0.50) | 3 675 542 (0.09) |

cTTR: centre time in therapeutic range; ICER: incremental cost-effectiveness ratio; INR: international normalised ratio; VKA: vitamin K antagonist

### Probabilistic Sensitivity Analyses

The incremental outcomes in terms of QALYs gained were plotted against incremental costs of apixaban versus other comparators on the cost-effectiveness planes for all 2000 iterations, shown in Figures 3a and 3b for the VKA-unsuitable and VKA-suitable populations, respectively. In the VKA-unsuitable population, the cost-effectiveness plane suggested that in the majority of iterations apixaban was more effective and more costly than aspirin and acenocoumarol. When compared to rivaroxaban, the cost-effectiveness plane suggested that apixaban was more effective and less costly in most of the iterations.

**Figure 3.** Results of Probabilistic Sensitivity Analyses for the VKA-unsuitable Population a) Cost-effectiveness plane for apixaban versus aspirin (incremental costs and QALYs)

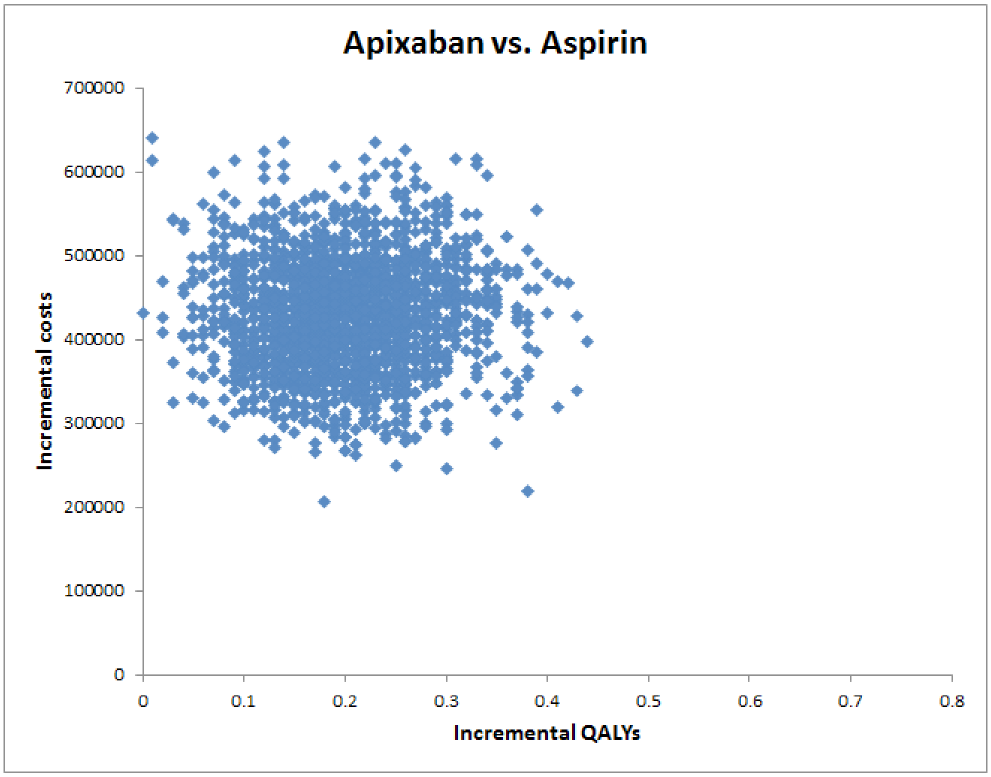

b) Cost-effectiveness acceptability curve for apixaban and aspirin (QALY)

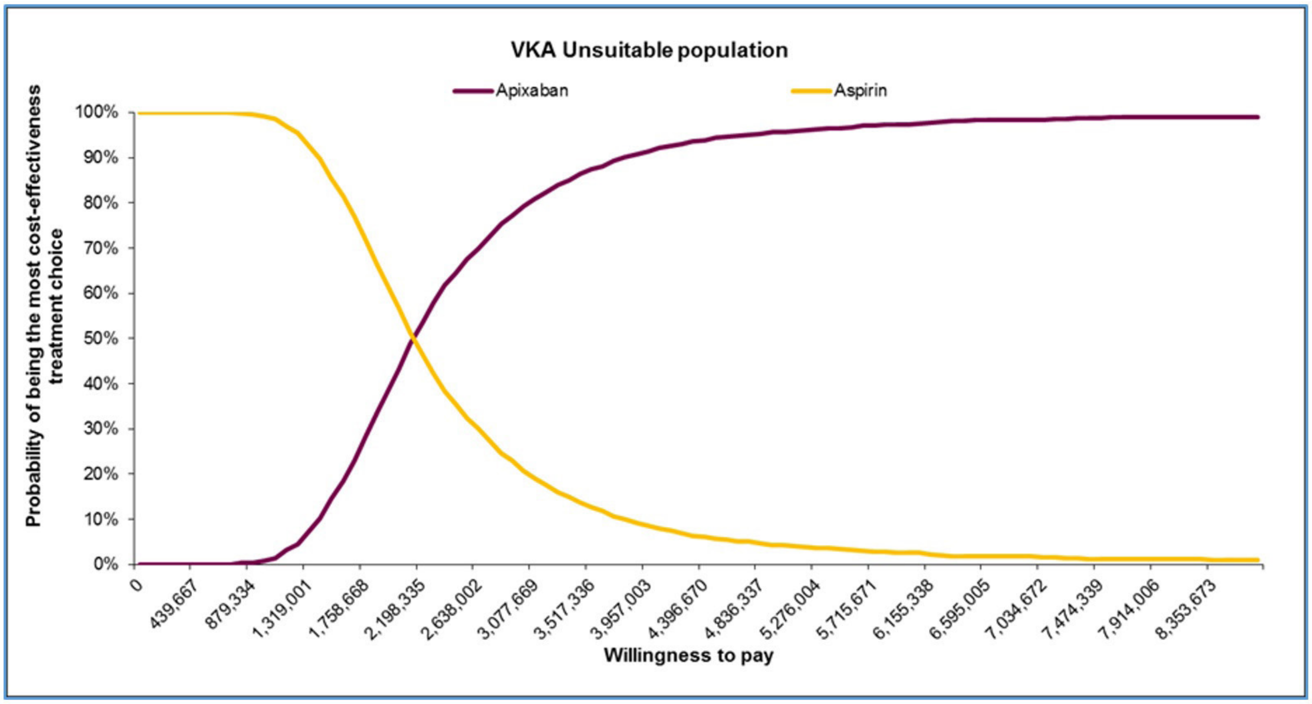

QALY: quality-adjusted life-year; VKA: vitamin K antagonist

When compared to aspirin, the treatment of VKA-unsuitable patients with apixaban was an optimal treatment choice, representing the maximum net benefit at a willingness to pay threshold above 2 198 335 DZD ($20 242, £13 800; based on exchange rates in 2015, where $1=DZD 108.6 and £1=DZD 159.3) per QALY (Figure 4a). At a willingness to pay threshold of 5 430 721 DZD (i.e., $50 000), apixaban was considered a cost-effective option in 96% of iterations when compared to aspirin. At a willingness to pay threshold of 4 779 190 DZD (i.e. £30 000) per QALY, apixaban was considered cost-effective in 94% of iterations. According to the CEAC for VKA-suitable patients (Figure 4b), apixaban was an optimal treatment choice over acenocoumarol and rivaroxaban at a willingness to pay threshold above 3 869 070 DZD ($35 627, £24 288; based on exchange rates in 2015, where $1=DZD 108.6 and £1=DZD 159.3) per QALY. Below this threshold, acenocoumarol was an optimal treatment option.

**Figure 4.** Results of Probabilistic Sensitivity Analyses for the VKA-suitable Population a) Cost-effectiveness plane for apixaban versus acenocoumarol and apixaban versus rivaroxaban (incremental costs and QALYs)

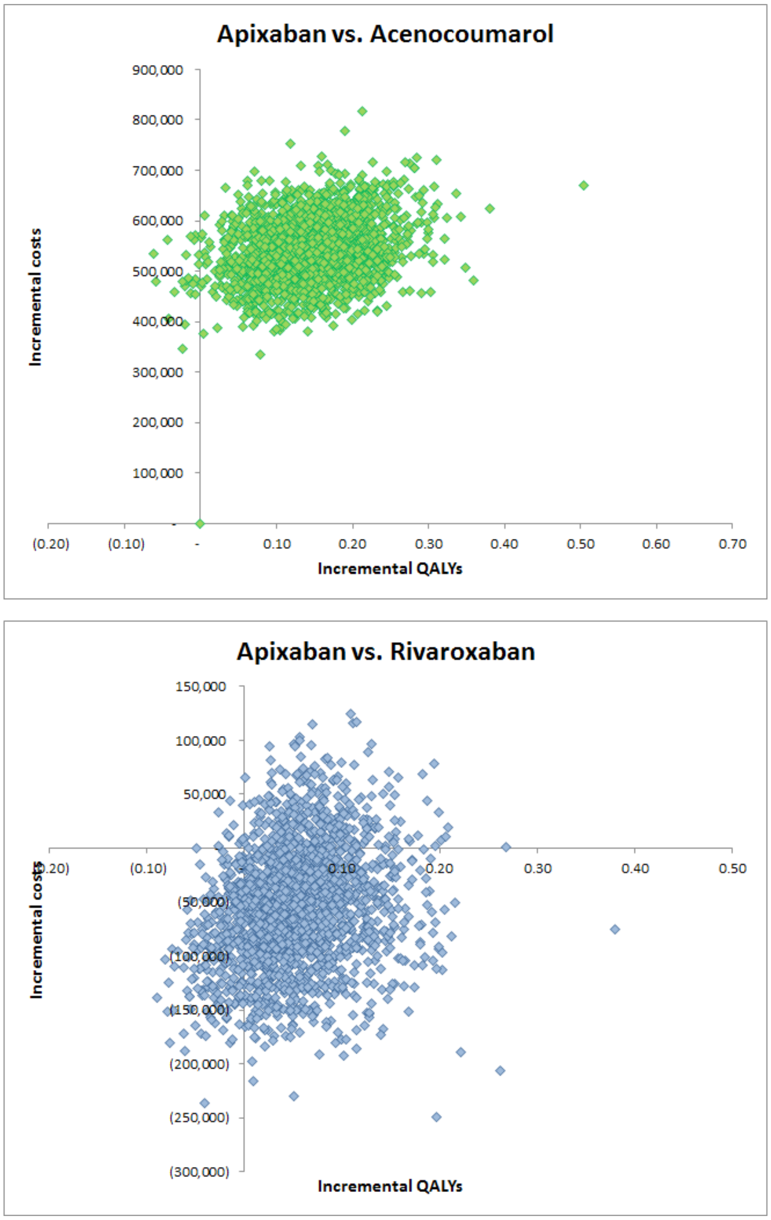

QALY: quality-adjusted life-year; VKA: vitamin K antagonist b) Cost-effectiveness acceptability curve for apixaban, acenocoumarol and rivaroxaban (QALY)

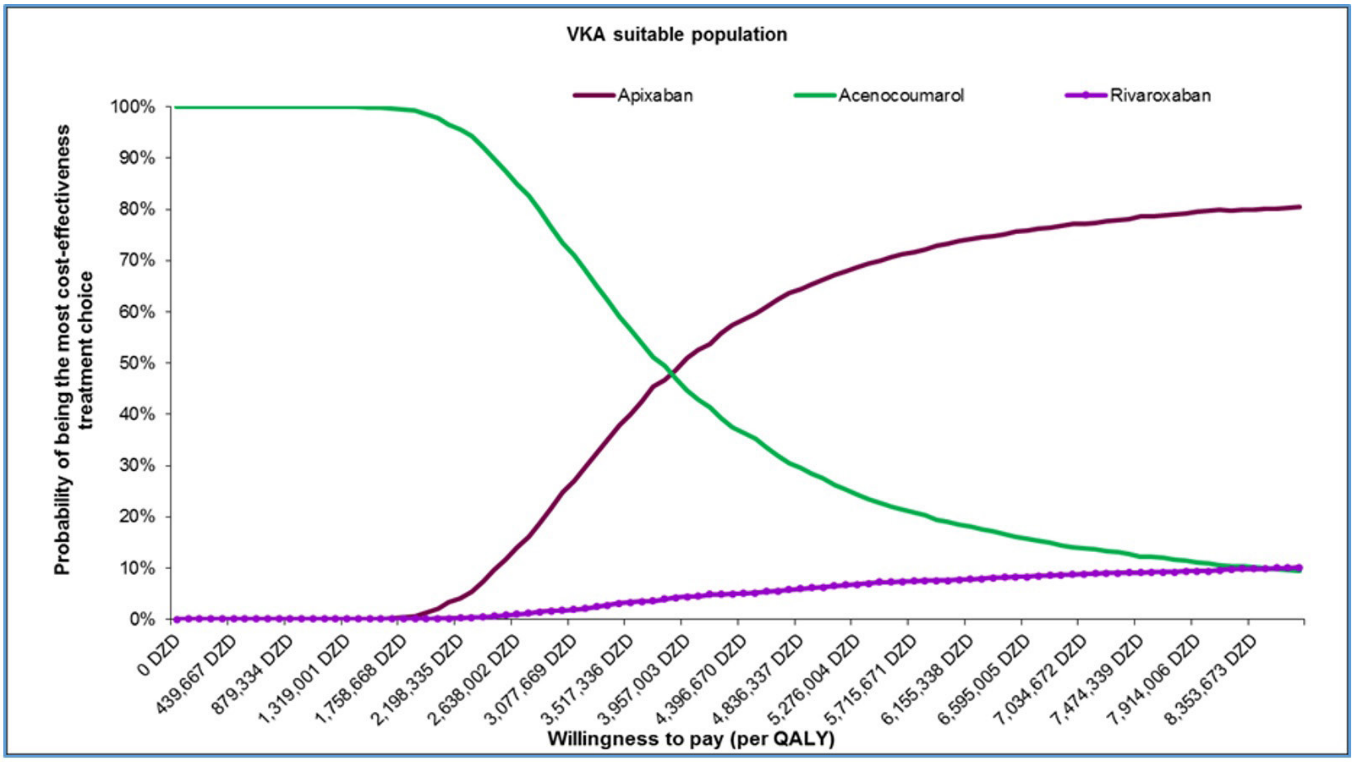

QALY: quality-adjusted life-year; VKA: vitamin K antagonist

## DISCUSSION

This study assessed the cost-effectiveness of apixaban compared to other available treatments, including aspirin, acenocoumarol and rivaroxaban, from an Algerian payer perspective. Among the patients suitable for VKA, fewer thromboembolic events and bleedings were estimated with apixaban compared to acenocoumarol and rivaroxaban, and these translated into LYs and QALYs gained. Apixaban was estimated to be a dominant treatment option over rivaroxaban, providing a higher number of QALYs at lower costs, while when compared with acenocoumarol, an ICER of 3 672 059 DZD ($37 054, £23 051) per QALY gained was estimated.

Among those unsuitable for VKA, although apixaban was estimated to slightly increase in the number of bleedings, it still led to LYs and QALYs gained due to a significant reduction in the number of thromboembolic events, with an estimated ICER of 2 061 863 DZD ($35 627, £24 288) per QALY gained.

Lower medical care costs were observed with apixaban due to the reduction in overall clinical events. Treatment costs increased with apixaban due to the higher acquisition cost compared to aspirin and acenocoumarol, and longer use with apixaban given the increased in life expectancy and lower discontinuation rates as observed in the clinical trials.

To date, our study is the first published economic evaluation assessing the cost-effectiveness of apixaban for stroke prevention in patients with NVAF from an Algerian payer's perspective. There are, however, some important limitations to these analyses. In Algeria, the standard anticoagulant used in patients eligible for VKA is acenocoumarol. Due to the unavailability of efficacy and safety evidence to link acenocoumarol to the apixaban clinical trial, the study used results extrapolated from clinical trials for warfarin. Although these may not reflect the true efficacy of acenocoumarol, findings from prior analyses and the use of this assumption in the previous cost-effectiveness studies support this assumption.[14-16] Another limitation of the current study relates to the unavailability of Algerian-specific utilities. The analysis employed utilities based on a UK EQ-5D catalogue.[12] Since these were taken from a European population, it is plausible to assume that they would be similar for the Algerian population. The same assumption was applied in other prior studies.[17-19] In addition, results from univariate sensitivity analysis, varying utility for each clinical event by its' 95% confidence intervals, demonstrated that the ICERs were altered by only less than 4%.

Previous cost-effectiveness analyses for apixaban have been conducted in Europe, the US and Latin America using different willingness to pay thresholds established in the countries involved in order to determine cost-effectiveness.[20] In Algeria, there is no such established threshold, therefore, these analyses considered threshold values that have been established in other countries, namely the US and the UK. An alternative approach for determining cost-effectiveness in low and middle income countries is the threshold of one-to-three times per capita income for averting a disability-adjusted life-year recommended by WHO.[21] Although this approach has been frequently adopted in prior studies, the lack of empirical evidence to support this rule has been widely criticised.[22,23] In addition, the outcomes assessed in the model used in this study included QALYs as opposed to DALYs, therefore, the WHO threshold was not considered in the present analyses. It is widely acknowledged that a broad range of factors are shaping decision making criteria especially in the context of low and middle income countries.[23,24] The authors believe that budgetary constraints and priorities set within the current political and institutional context in Algeria should ultimately be taken into account for appropriate decision making.

## CONCLUSION

The study demonstrated that apixaban is a cost-effective choice for stroke prevention in patients with AF compared to acenocoumarol and rivaroxaban in the VKA-suitable population and compared to aspirin in the VKA-unsuitable population.

## DISCLOSURE

This study was funded by Pfizer. Thitima Kongnakorn and Evie Merinopoulou are full-time employees of Evidera and served as paid consultants to Pfizer in association with conducting this study and with the development of this manuscript. Mohamed Said Bettayeb and Sid Ahmed Kherraf are full-time employees of Pfizer with ownership in stocks.
